# A giant isolated primary splenic hydatidosis: A case report

**DOI:** 10.1016/j.amsu.2022.104863

**Published:** 2022-11-14

**Authors:** Rajan Kumar Sah, Biki Kumar Sah, Chau Thi Minh Dang, Vivek Kumar Sah

**Affiliations:** a Manipal College of Medical Sciences, Pokhara, Nepal; b B.P. Koirala Institute of Health Sciences, Dharan, Nepal; c B.P. Koirala Institute of Health Sciences, Dharan, Nepal; d Universal College of Medical Sciences and Teaching Hospital, Bhairahawa, Nepal

**Keywords:** Hydatid disease, Splenic hydatid, Ultrasonography, Case report

## Abstract

**Introduction:**

and importance: Hydatid cyst disease is caused by Echinococcus tapeworm and is one of the major health problems in endemic regions like Nepal. The cases of splenic hydatidosis are quite rare and giant isolated primary splenic hydatidosis is even rarer. The patients present with vague symptoms or no symptoms at all. Here we report a case of isolated splenic hydatid cyst. So, we should think the differential diagnosis of splenic hydatidosis in any abdominal case of endemic regions.

**Case presentation:**

A 27-year-old female presented with left-side abdominal pain for the past 7 months without any particular attraction. Abdominal ultrasound showed a well-defined cystic mass on the upper pole with low-level internal floating debris. Contrast Enhanced CT scan showed well defined cystic lesion measuring about 10.8 × 9.6 × 8.5 cm in the upper pole of the spleen with an exophytic component and minimal homogenous wall enhancement. Laparoscopic Splenectomy was done and albendazole for 3 weeks was prescribed after all the patient was completely normal.

**Clinical discussion:**

In this case, the optimal treatment of giant isolated splenic hydatidosis was splenectomy and prescription of albendazole.

**Conclusion:**

We believe in any abdominal case of the endemic region, the hydatid cyst of the spleen should be taken as one of the differential diagnoses and should be managed appropriately before the complication arises.

## Highlights

• Splenic HC is often asymptomatic.
• Intraperitoneal rupture of splenic HC can cause life-threatening anaphylaxis.
• Diagnosis of splenic HC requires a high index of suspicion.
• Abdominal ultrasound and computed tomography are modalities of choice for diagnosis.
• Standard treatment for splenic HC is splenectomy.

## 1 Introduction

Hydatid cyst results from infection by *Echinococcus* tapeworm; most commonly being *Echinococcus granulosus* and least common *Echinococcus alveolaris* [1]. The definitive host is carnivorous (e.g.: dogs, foxes, cats) and the intermediate host is sheep. Humans are accidental hosts and infection occurs by ingesting food contaminated with Echinococcus egg [2]. Echinococcosis is globally distributed though it is endemic in parts of Central Asia, Argentina, Peru, China, South America, and North Africa. Hydatid cyst can occur anywhere although it is commonly found in the liver (60–70%) or lung (30%) and is rarely encountered in the kidney, spleen, breast, thyroid, bone, and pancreas [3]. Primary infestation of the spleen usually takes place when the ova bypass hepatic and pulmonary capillary bed circulation and enters systemic circulation forming cysts in the spleen. Secondary infections, fistulation to adjacent organ, rupture into peritoneal cavity complications may arise. A life-threatening complication of a systemic anaphylactic reaction may arise due to a traumatic or spontaneous rupture of a hydatid cyst [3]. In this case report, we describe a rare case of a giant isolated primary splenic hydatid cyst in a 27-year-old female patient This case report has been reported in line with the SCARE Criteria [4].

## 2 Case report

**Fig. 1:**
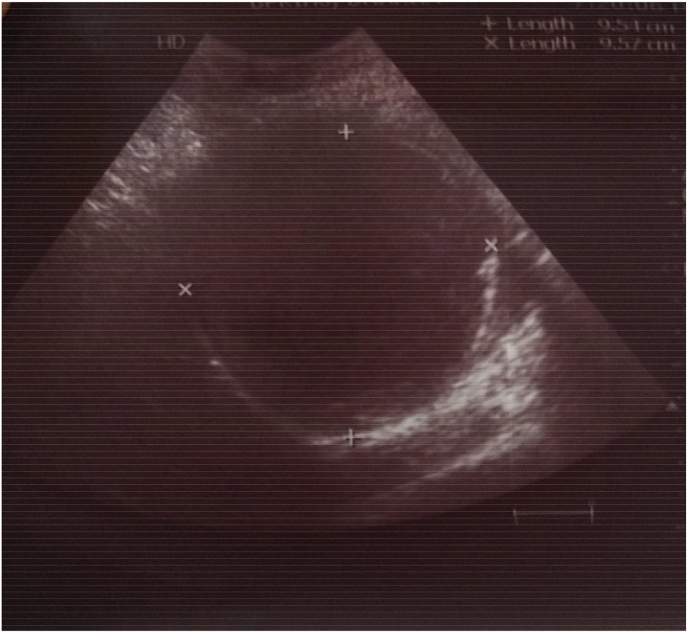
A well-defined cystic mass with low-level internal floating debris and echogenic strap measuring 9.5 × 9.6 cm noted at the dependent portion of spleen.

**Fig. 2:**
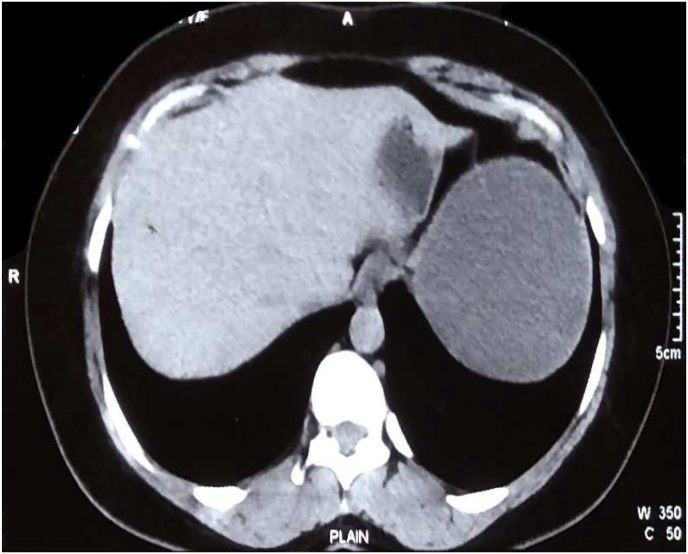
A well-defined cystic lesion measuring about 10.8 × 9.6 × 8.5 cm in the upper pole of spleen with exophytic component and minimal homogenous wall enhancement.

A 27-year-old female recently married with no comorbidities presented to the surgical outpatient department with a complaint of abdominal pain in the left hypochondrium region for 7 months. The pain was gradual in onset, dull aching in character. She has normal bowel and bladder habits with no history of jaundice, fever, vomiting, and significant weight loss. There is no any relevant drug history, family history, genetic information and psychosocial history related with the case. On general examination, she had a GCS score of 15 and her vitals were normal. On per abdominal examination, the abdomen was soft and non-tender in palpation. On auscultation abdominal sounds were present. The laboratory investigations showed hemoglobin, Red Blood Cells (RBCs), White Blood Cells (WBCs), Platelets, Packed Cell Volume (PCV), Prothrombin Time (PT), International normalized ratio (INR), and Partial Thromboplastin Time (PTT), creatinine, electrolytes, and liver function tests were in the normal range. Abdominal Ultrasound showed a well-defined cystic mass with low-level internal floating debris and an echogenic strap measuring 9.5 cm × 9.6 cm was noted at the dependent portion, the mass was noted in the upper pole of the spleen (Fig. 1). It was confirmed by a contrast-enhanced CT scan of the abdomen which showed well defined cystic lesion measuring about 10.8 × 9.6 × 8.5 cm3 in the upper pole of the spleen with exophytic component and minimal homogenous wall enhancement. No calcification, septation, and enhancing solid components were seen. (Fig. 2). Laparoscopic splenectomy was performed by Dr. Suresh Parsad Sah with standard four ports. A single cyst of size 11 × 10 × 8 cm3 was present on the upper pole of the spleen. Liver and rest viscera were normal. On the cut section, there was hydatid sand and fluid around 1 L in amount. Patient was compatible with the procedure and there was blood loss of around 100ml. The postoperative period was uneventful and the patient was discharged on postoperative day 6. The postoperative course was uneventful and 400mg of albendazole twice a day was given for three weeks. Patient was satisfied with the laparoscopic procedure done as she felt less pain and less hospital stay and also she was able to resume her daily activities within a week after being discharged. After three weeks patient visited the hospital for follow-up. Echinococcus IgG ELISA serology test was done and it was negative. The patient completely recovered with no complaints.

## 3 Discussion

Echinococcal infection in humans is often caused by four species, among which *E. granules* and *E. multilocularis* are the most popular. These organisms are the etiologies of cystic echinococcosis (CE), also known as cystic hydatid disease, and alveolar echinococcosis (AE), correspondingly. E. vogeli and E. eligarthrus, which generate polycystic echinococcosis, are less commonly seen [5].

CE can occur in any organ, among which the liver (75%), lung (15.4%), and spleen (5.1%) were the most dominant [6]. For the population in endemic areas, 50–80% of splenic cysts are caused by Echinococcus [7]. Interestingly, isolated splenic involvement represents only 2.04% of abdominal HC and 0.7% of infected patients [8,9]. This rarity can be explained by the fact that to cause a primary splenic infestation via the arterial route, Echinococcal oncospheres have to pass the hepatic and pulmonary filters. Although retrograde venous route via portal circulation bypassing the liver and lung was reported, this passage is infrequent [8,10,11].

Meanwhile, concomitant hepatic or pulmonary hydatid cyst (HC) is often reported in 20–30% of cases, representing systemic dissemination. Additionally, ruptured hepatic HC spreads parasites intraperitoneally causing secondary splenic HC [11].

As splenic HC expands slowly with a rate of 0.3 cm–1 cm per year, it is often asymptomatic and detected accidently on physical examination as an abdominal mass in the left hypochondrium (30%). Patients can also present with abdominal pain (25%), discomfort, or compressive symptoms such as dyspepsia, dyspnea, and constipation [8,11].

Regarding complications, intraperitoneal rupture of splenic HC, which could be spontaneous or trauma-induced, can cause life-threatening anaphylaxis due to slippage of protoscolex mass in the left hypochondrium (30%). Perforation into the colon can manifest as haematochezia, even massive gastrointestinal bleeding. Moreover, a splenothoracic fistula has also been reported. Later, pericystic inflammation is followed by adhesion to a nearby organ. Other complications are secondary infection of splenic HC and splenic atrophy [10].

Diagnosis of splenic HC requires a high index of suspicion, especially in endemic areas. It requires a combination of clinical findings, imaging, and immunological tests. The key point is to distinguish from other splenic cystic formations, including splenic abscesses or hematomas, epidermoid cysts, post-traumatic pseudo cysts and neoplasms [11].

Abdominal ultrasonography and computed tomography are usually the modalities of choice for diagnosis procedures [9]. With a sensitivity of 90–95%, ultrasound is the first radiological test to be chosen when a splenic HC is suspected. The World Health Organization's Informal Working Group for Echinococcosis (WHO-IWGE) has classified the ultrasound appearance of CE into 5 types. The most popular image for splenic HC is a solitary, anechoic smooth round cyst, which should be differentiated from a benign cyst [9,11]. However, CT has higher sensitivity of 90–100% and should be considered for suspected associated intra-abdominal localizations, complications, presurgical evaluations or detecting recurrence. A combination of US-CT has been suggested thanks to the diagnostic accuracy of nearly 100% [9,10].

Serology test has a limited role in diagnosis but could be useful for follow-up [9]. The Echinococcus IgG ELISA test has a sensitivity and specificity varying from 72% to 96%, and 40.8% and 97%, respectively [12].

Standard treatment for splenic HC is splenectomy, including total splenectomy, partial splenectomy, cyst enucleation and unroofing with omentoplasty, given the low efficacy of the medication. Total splenectomy has a low recurrence rate and should be indicated for large HC in which splenic parenchyma has been drastically reduced and brittle to prevent ruptured complications [9,11]. At the same time, total splenectomy carries a risk of overwhelming post-splenectomy sepsis (OPSI) with an incidence between 0.9 and 60% and a mortality of over 50%. 1.9% of adults and 4% of children were reported with sepsis-related deaths after splenectomy [11]. Therefore, spleen-preserving procedures are favored for children or peripherally located cysts, which occupy <50%–75% of the parenchyma [9]. In the present case, the patient was treated with laparoscopic total splenectomy.

Perioperative management includes anti-helminthic drugs and vaccinations. Medical regiments are Albendazole 10–15 mg/kg/day for one month or Mebendazole 40–50 m/kg/day for 3–6 months after surgery, combined with Praziquantel 40 mg/kg/wk for 2 weeks during pre and postoperative period. Nevertheless, Albendazole is preferred due to its superior bioavailability. Plus, Hib and meningococcus C conjugate vaccines should be administered at least 2 weeks before or 2 weeks post-splenectomy [10,11].

Percutaneous aspiration irrigation and reaspiration (PAIR) could be indicated for patients who refuse surgery or have greater anesthetic risk. Still, it is associated with prolonged follow-up and should be avoided in calcified or abscessed cysts [11].

## 4 Conclusion

Despite advances in medical sciences, splenic hydatidosis has been a diagnostic challenge. The various investigation cannot always reach the diagnosis. It is important to consider the hydatid cyst in one of the differential diagnoses in abdominal cysts and must be in splenic cysts if the clinical condition justifies in endemic regions. Standard splenectomy and prophylaxis with albendazole are suggested.

## Ethical approval

The patient was informed, and we have acquired her consent for this publication.

## Source of funding

None.

## Author contributhon

Writing the paper: Dr. Rajan Kumar Sah and Dr. Chau Thi Minh Dang, Surgical treatment for this patient: Biki Kumar Sah, Study design: Dr. Rajan Kumar Sah, Data interpretation: Vivek Kumar Sah.

## Trail registry number

N/A.

## Garantor

Biki Kumar Sah.

## Provenance and peer review

Not commissioned, externally peer reviewed.

## Declaration of competing interest

None.
